# Effects of isobaric spinal anesthesia combined with different nerve block techniques on postoperative pain and complications in elderly patients undergoing proximal femoral nail antirotation: a retrospective observational study

**DOI:** 10.3389/fmed.2026.1813736

**Published:** 2026-10-12

**Authors:** Jingjing Li, Ning Ma, Yuan Zhao, Na Liang, Yanbin Zhao

**Affiliations:** 1 Department of Medical Records and Statistics, People’s Hospital of Ningxia Hui Autonomous Region, Ningxia Medical University, Yinchuan, Ningxia, China; 2 Department of Anesthesiology, Yinchuan Second People’s Hospital, Yinchuan, Ningxia, China; 3 Department of Neurosurgery, People’s Hospital of Ningxia Hui Autonomous Region, Ningxia Medical University, Yinchuan, Ningxia, China

**Keywords:** complications, elderly patients, nerve block, pain, proximal femoral nail antirotation, spinal anesthesia

## Abstract

**Objective:**

To explore the impacts of isobaric spinal anesthesia combined with different nerve blocks on postoperative pain, complications, satisfaction and perioperative comfort in elderly patients undergoing proximal femoral nail antirotation(PFNA).

**Methods:**

A retrospective analysis was conducted on elderly PFNA patients enrolled from 2019 to 2021 in the Department of Traumatic Orthopedics at the People’s Hospital of Ningxia Hui Autonomous Region. Patients were divided into four groups: group S(simple isobaric spinal anesthesia), group S + FNB(spinal anesthesia plus femoral nerve block), group S + LFCN(spinal anesthesia plus lateral femoral cutaneous nerve block), group S + FICB(spinal anesthesia plus fascia iliaca compartment block). Primary outcomes were dynamic VAS scores at different postoperative time points, while secondary outcomes comprised postoperative complications and pain management indicators. All data were collected and statistically compared.

**Results:**

A total of 191 elderly patients who underwent PFNA met the inclusion criteria. In group S, VAS scores on postoperative days 2 and 3 were lower than those at 1 day preoperatively, whereas in groups S + FNB, S + LFCN, and S + FICB, VAS scores on postoperative days 1, 2, and 3 were lower than preoperative values. In all four groups, VAS scores on postoperative days 2 and 3 were lower than those on postoperative day 1 (*P* < 0.05). On postoperative day 1, VAS scores in groups S + FNB and S + LFCN were lower than those in group S, while VAS scores in group S + FICB were significantly lower than those in groups S + FNB, S + LFCN, and S on postoperative days 1, 2, and 3 (*P* < 0.05). The incidence of postoperative nausea, vomiting, and pruritus was lower in group S + FICB than in group S (*P* < 0.05). The dose of sufentanil required for rescue analgesia was lower in groups S + LFCN and S + FICB than in group S, and lower in group S + FICB than in groups S + FNB and S + LFCN (*P* < 0.05). The number of patients requiring postoperative rescue analgesia was lower in group S + FICB than in groups S + FNB, S + LFCN, and S (*P* < 0.05). Postoperative satisfaction was higher in group S + FICB than in group S (*P* < 0.05).

**Conclusion:**

Preoperative isobaric spinal anesthesia combined with fascia iliaca compartment block was associated with better postoperative pain relief, reduced adverse reactions, and improved perioperative comfort in elderly patients, and was more effective than other anesthesia strategies.

## 1 Introduction

Population aging has become an increasingly serious public health challenge in China. Data from the Seventh National Population Census indicate that the population aged 60 years and older has reached 264 million, accounting for 18.7% of the total population (1). Among elderly individuals with chronic diseases, osteoporosis and osteoporotic fragility fractures, as age-related conditions, have attracted increasing attention (2, 3). Femoral neck fracture (FNF) is one of the most common fractures encountered in clinical practice. Epidemiological data (4) show that among every 100,000 patients with FNF, the average incidence in individuals older than 60 years is approximately 89.5%. Following FNF, 28.8% of patients develop lower-extremity deep vein thrombosis due to prolonged immobilization (5), 12%–86% experience avascular necrosis of the femoral head as a result of disrupted blood supply at the fracture site (6), and some elderly patients with severe conditions may even die (7).

Conservative treatment does not effectively relieve pain in patients with FNF, and surgical intervention remains the preferred therapeutic approach. In recent years, proximal femoral nail antirotation fixation has been widely applied in the treatment of FNF because of its advantages, including technical simplicity, minimal surgical trauma, reduced blood loss, and stable internal fixation (8). However, elderly patients frequently present with multiple comorbidities, and postoperative pain stimulation may trigger delirium, cognitive decline, depression, and cardiovascular or cerebrovascular events. Therefore, an appropriate and effective anesthetic strategy is not only essential for optimizing the surgical process but also a key determinant of surgical success.

Preemptive analgesia is an important component of perioperative pain management and has been shown to significantly reduce postoperative pain, decrease opioid consumption, and lower the incidence of nausea and vomiting (9). At present, various nerve block techniques are widely used for preemptive analgesia in elderly patients undergoing PFNA. However, robust evidence comparing different anesthetic strategies with respect to patient comfort and postoperative complications remains limited. Accordingly, this retrospective study was conducted to further investigate which compound anesthesia regimen best reduces postoperative pain and complication rates, so as to provide a reference for clinical anesthesiologists aiming to improve satisfaction and perioperative comfort in elderly PFNA patients.

## 2 Materials and methods

### 2.1 Study design

This study employed a retrospective analysis based on routinely collected clinical data. Elderly patients who underwent PFNA in the Department of Traumatic Orthopedics at the People's Hospital of Ningxia Hui Autonomous Region between October 2019 and October 2021 were retrospectively identified through the electronic medical record management system. This study was reviewed and approved by the Ethics Committee of the People's Hospital of Ningxia Hui Autonomous Region (approval number: 2022-LL-109), and the requirement for informed consent was waived owing to the retrospective design.

### 2.2 Participants

All eligible participants were identified retrospectively from the electronic medical record database of People's Hospital of Ningxia Hui Autonomous Region. Inclusion criteria were:(1) age between 60 and 80 years, (2) American Society of Anesthesiologists (ASA) physical status classification II–III, (3) body mass index (BMI) between 18.5 and 24 kg/m^2^, (4) imaging-confirmed diagnosis of femoral neck fracture (FNF), (5) first-time PFNA performed under isobaric spinal anesthesia with bupivacaine–fentanyl (0.75% bupivacaine 15 mg combined with fentanyl 50 μg, total volume 3 mL), (6) administration of different nerve block techniques using 20–30 mL of 0.25%–0.375% ropivacaine, (7) use of postoperative intravenous patient-controlled analgesia (PCIA), with a primary formulation consisting of sufentanil 100–120 μg diluted with normal saline to a total volume of 120 mL.

Patients were excluded if they had (1) presence of severe chronic diseases, including hypertension [systolic blood pressure ≥ 180 mmHg and/or diastolic blood pressure ≥ 110 mmHg was defined as severe hypertension (grade 3 hypertension)], coronary heart disease(left main coronary artery stenosis ≥ 50%, stenosis ≥ 70% in any single major epicardial coronary artery, or stenosis ≥ 70% in two or more major coronary arteries), or diabetes mellitus [diabetes complicated with acute or chronic complications, or glycated hemoglobin (HbA1c) ≥ 9.0%], (2) concomitant multiple trauma or severe traumatic brain injury, (3) incomplete postoperative follow-up data, (4) long-term use of opioid or sedative medications, (5) absence of postoperative PCIA use, (6) old femoral neck fractures or infection at the fracture site, (7) difficulty with neuraxial anesthesia puncture, including patients with ankylosing spondylitis, prior lumbar spine surgery, or anatomical abnormalities.

The nerve-block technique administered to each patient was determined by the attending anesthesiologist, with reference to hospital anesthesia protocols and individual patient-specific factors including preoperative physical status, comorbidities, and pain conditions. Patients were retrospectively divided into four groups on the basis of the actually performed nerve-block procedures extracted from medical records. Group S (isobaric spinal anesthesia with bupivacaine-fentanyl): Patients were placed in the lateral decubitus position with the affected limb uppermost. After skin disinfection and draping, spinal puncture was performed at the L_3–4_ interspace using a 25-G spinal needle guided by a 20-G introducer. After the appearance of clear cerebrospinal fluid, an isobaric solution consisting of 0.75% bupivacaine 15 mg combined with fentanyl 50 μg was injected into the subarachnoid space in a cephalad direction at a rate of 0.3 mL/s (10). Group S + FNB (spinal anesthesia combined with femoral nerve block): A high-frequency ultrasound probe (8–12 MHz) was used for scanning. With the femoral artery identified as the reference point, the probe was moved cephalad until the proximal branching of the femoral artery was visualized. The femoral nerve was located lateral to the femoral artery. Using an in-plane technique, 0.375% ropivacaine was injected, with 10 mL administered above and 10 mL below the femoral nerve (11). Group S + LFCN (spinal anesthesia combined with lateral femoral cutaneous nerve block): A high-frequency ultrasound probe (8–12 MHz) was placed transversely inferior to the anterior superior iliac spine. The sartorius muscle and tensor fasciae latae were identified, and the lateral femoral cutaneous nerve was located on the lateral surface of the sartorius muscle. Subsequently, 20 mL of 0.375% ropivacaine was injected (12). Group S + FICB (spinal anesthesia combined with fascia iliaca compartment block): A linear ultrasound probe was positioned with one end over the anterior superior iliac spine and the other directed toward the umbilicus, with gentle pressure applied toward the umbilical side. From lateral to medial, the anterior superior iliac spine, iliacus muscle, iliac fascia, deep circumflex iliac artery, transversus abdominis muscle, and internal oblique muscle were visualized. Using an in-plane technique, the needle was advanced from the lateral side of the probe until the tip reached the space between the iliacus muscle and the iliac fascia. After confirming appropriate needle placement and negative aspiration, 20 mL of 0.375% ropivacaine was injected (13).

### 2.3 Variables

All study variables were retrospectively obtained from the electronic medical records of People’s Hospital of Ningxia Hui Autonomous Region.

The primary outcome: comparison of dynamic visual analog scale (VAS) scores at different time points: at 1 day preoperatively and on postoperative days 1, 2, and 3 (the VAS score is defined as 0 cm = no pain, 10 cm = the worst imaginable pain).

The secondary outcome: (1) Postoperative complications: incidence of nausea and vomiting, pruritus, urinary retention, agitation, shivering, and dyspnea. (2) Postoperative pain management indicators: additional analgesic dosage, number of rescue analgesia cases (If postoperative VAS score ≥ 4, rescue analgesia was administered by an anesthesiologist as an intravenous injection of sufentanil 5 ug), and postoperative analgesia satisfaction (including satisfied, acceptable, dissatisfied).

The following measures were implemented to ensure the objectivity and accuracy of follow-up data: (1) all PFNA procedures were performed by experienced orthopedic surgeons, and all isobaric spinal anesthesia and nerve block techniques were administered by experienced anesthesiologists, all nerve blocks were performed under ultrasound guidance 10 min after the implementation of isobaric spinal anesthesia. (2) cases were strictly screened by the principal investigator and research staff according to the inclusion and exclusion criteria to ensure data reliability and authenticity. (3) data processing and statistical analyses were conducted strictly in accordance with standardized statistical software procedures to ensure accuracy of the results.

### 2.4 Study size

A total of 191 patients met all inclusion criteria after screening 200 hospitalized cases. The sample size was determined by the number of eligible patients available within the study period.

### 2.5 Statistical analysis

Statistical analyses were performed using SPSS version 25.0. Continuous variables were expressed as mean ± standard deviation and were analyzed using one-way ANOVA or repeated-measures analysis of variance. Continuous variables that were not normally distributed are presented as median with interquartile range [*M* (*P_25_, P_75_*)] and were analyzed using the *Kruskal–Wallis H* test, with pairwise comparisons conducted using the *Bonferroni* method. Categorical variables are presented as counts (*N*) or percentages (%) and were analyzed using the Chi-square test or Fisher's exact test. A *P* < 0.05 was considered statistically significant.

## 3 Results

### 3.1 Baseline data

After strict application of the inclusion and exclusion criteria, a total of 191 elderly patients were included in the final analysis. According to the anesthetic strategy applied, patients were retrospectively divided into four groups: group S (*N* = 48), group S + FNB (*N* = 47), group S + LFCN (*N* = 48), and group S + FICB (*N* = 48) (Figure 1). Comparisons of age, sex distribution, body mass index, American Society of Anesthesiologists physical status, preoperative comorbidities, intraoperative anesthesia time, operative time, total intraoperative fluid input and output, the dosage of ropivacaine, the rate of atropine and norepinephrine use among groups S, S + FNB, S + LFCN, and S + FICB showed no statistically significant differences (all *P* > 0.05) (Table 1).

**Figure 1:**
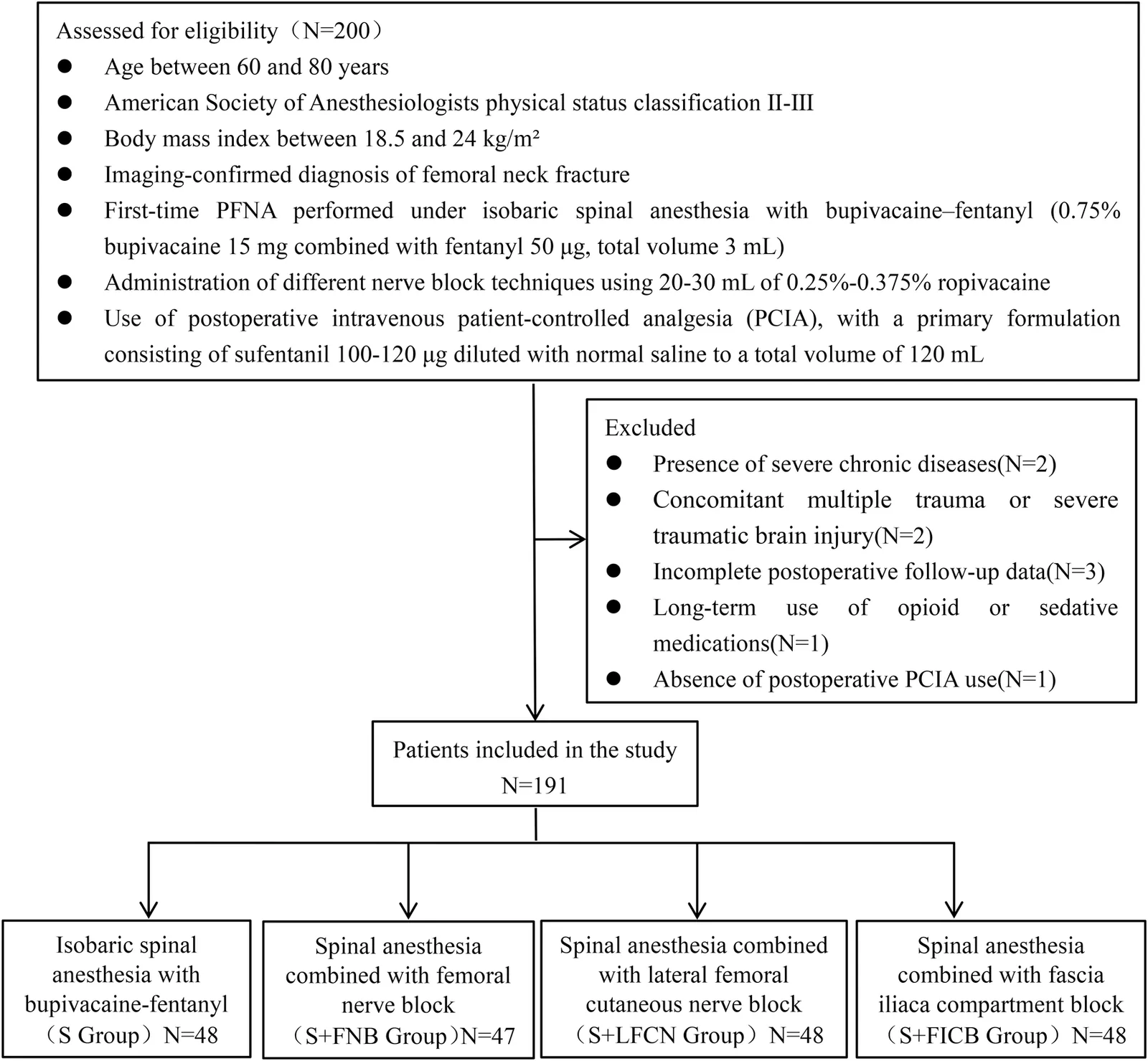
Flow diagram of patient selection and exclusion criteria.

**Table 1:** Baseline characteristics of patients.

Variable	S Group (*N* = 48)	S + FNB Group (*N* = 47)	S + LFCN Group (*N* = 48)	S + FICB Group (*N* = 48)	*H/χ2*	*P*
Age(years)	72 (67, 73.8)	72 (66, 75)	73.5 (71.3, 75.8)	72 (68, 74)	6.053	0.109
Sex					3.321	0.345
Male	22（45.8）	14（29.8）	16（33.3）	15（31.3）		
Female	26（54.2）	33（70.2）	32（66.7）	33（68.7）		
BMI(kg/m^2^)	21.6 (19.8, 24)	22.3 (20, 24)	21.94 (19.8, 24)	22.0 (20.3, 22.8）	1.742	0.628
ASA physical status					3.889	0.274
II	39（81.3）	32（68.1）	33（68.8）	31（64.6）		
III	9（18.7）	15（31.9）	15（31.2）	17（35.4）		
Preoperative comorbidities						
Hypertension	19（39.6）	20（42.6）	28（58.3）	26（54.2）	7.914	0.244
Coronary heart disease	10（20.8）	11（23.4）	5（10.4）	7（14.6）	3.588	0.310
Diabetes mellitus	8（16.7）	10（21.3）	14（29.2）	13（27.1）	5.668	0.461
Intraoperative anesthesia time(min)	107 (87, 133.8)	110 (80, 144)	100 (90, 123)	92.5 (80, 124.5)	1.490	0.685
Operative time(min)	86.5 (64.3, 119)	90.0 (70, 190)	85.0 (70, 130)	72.5 (60, 104.5)	3.162	0.367
Intraoperative fluid input(mL)	1900（1250, 3237.5）	2200（1750, 3400）	2200（1950, 3387.5）	2150（1500, 3462.5）	5.380	0.146
Intraoperative fluid output(mL)	600 (350, 900)	550 (200, 900)	600 (132.5, 900)	400 (162.5, 700)	3.071	0.381
The dosage of ropivacaine	25.94 ± 4.69	23.94 ± 4.54	25.21 + 4.94	25.42 ± 4.71	1.533	0.207
The rate of atropine use	12（25）	8（17）	6（12.5）	3（6.3）	7.201	0.066
The rate of norepinephrine use	9（18.8）	6（12.8）	5（10.4）	3（6.3）	3.735	0.292

Data are presented as *N*(%), and compared using Chi-square test; data are presented as median (interquartile range), and compared using *Kruskal–Wallis H* test; data are presented as mean ± SD, and compared using One-way ANOVA. S Group, bupivacaine-fentanyl isobaric spinal anesthesia; S + FNB Group, spinal anesthesia + femoral nerve block; S + LFCN Group, spinal anesthesia + lateral femoral cutaneous nerve block; S + FICB Group, spinal anesthesia + fascia iliaca compartment block. BMI, Body Mass Index; ASA, American Society of Anesthesiologists.

### 3.2 Evaluation of postoperative analgesic effect

#### 3.2.1 Comparison of VAS scores at different time points

Repeated-measures analysis of variance revealed a significant interaction effect between nerve block technique and time on VAS scores (*F_interaction_* = 11.924, *P_interaction_* < 0.001, partial *η^2^* = 0.161). A significant main effect of nerve block technique on VAS scores was also observed (*F_between groups_* = 23.367, *P_between groups_* < 0.001, partial *η^2^* = 0.273), as well as a significant main effect of time *(F_time_* = 217.722, *P_time_* < 0.001, partial *η^2^* = 0.538). In group S, VAS scores on postoperative days 2 and 3 were significantly lower than those at 1 day preoperatively (*P* < 0.05). In groups S + FNB, S + LFCN, and S + FICB, VAS scores on postoperative days 1, 2, and 3 were significantly lower than preoperative values (*P* < 0.05). In addition, VAS scores on postoperative days 2 and 3 were significantly lower than those on postoperative day 1 in all four groups (*P* < 0.05). Between-group comparisons showed that on postoperative day 1, VAS scores in groups S + FNB and S + LFCN were significantly lower than those in group S (*P* < 0.05). Furthermore, VAS scores in group S + FICB were significantly lower than those in groups S + FNB, S + LFCN, and S on postoperative days 1, 2, and 3 (*P* < 0.05) (Tables 2–4).

**Table 2:** Repeated-measures analysis of VAS scores among the four groups at different time points.

Time	S Group (*N* = 48)	S + FNB Group (*N* = 47)	S + LFCN Group (*N* = 48)	S + FICB Group (*N* = 48)	*F*	*P*	partial *η*2
D0	3.07 ± 0.98	3.21 ± 1.60	3.28 ± 1.15	3.31 ± 1.12	F_interaction_ = 11.924 F_between groups_ = 23.367 F_time_ = 217.722	P_interaction_ < 0.001 P_between groups_ < 0.001 P_time_ < 0.001	0.161 0.273 0.538
D1	3.17 ± 0.93	2.00 ± 0.74^c^^,^^d^	2.21 ± 0.77^c^^,^^d^	1.15 ± 0.45^a^^,^^b^^,^^c^^,^^d^			
D2	1.53 ± 0.6^d^^,^^e^	1.52 ± 0.6^d^^,^^e^	1.65 ± 0.7^d^^,^^e^	0.96 ± 0.48^a^^,^^b^^,^^c^^,^^d^^,^^e^			
D3	1.51 ± 0.4^d^^,^^e^	1.51 ± 0.6^d^^,^^e^	1.63 ± 0.7^d^^,^^e^	0.83 ± 0.43^a^^,^^b^^,^^c^^,^^d^^,^^e^			

Data are presented as mean ± SD, and compared using repeated-measures analysis of variance. S Group, bupivacaine-fentanyl isobaric spinal anesthesia; S + FNB Group, spinal anesthesia + femoral nerve block; S + LFCN Group, spinal anesthesia + lateral femoral cutaneous nerve block; S + FICB Group, spinal anesthesia + fascia iliaca compartment block. D0, the day before surgery; D1, postoperative day 1; D2, postoperative day 2; D3, postoperative day 3. A *P* < 0.05 was considered statistically significant. Compared with Group S + FNB.

a *P* *<* 0.05; Compared with Group S + LFCN.

b *P* *<* 0.05; Compared with Group S.

c *P* *<* 0.05; Compared with D0.

d *P* *<* 0.05; Compared with D1.

e *P* *<* 0.05.

**Table 3:** Simple effects analysis of time in four groups of VAS scores.

Group	Mean difference	Standard error	*P*	95% confidence interval
S Group				
D0-D1	−0.100	0.208	0.632	（−0.519–0.318）
D0-D2	1.538^a^	0.165	< 0.001	（1.206–1.870）
D0-D3	1.556^a^	0.171	< 0.001	（1.212–1.899）
D1-D2	1.638^a^	0.171	< 0.001	（1.293–1.983）
D1-D3	1.656^a^	0.150	< 0.001	（1.353–1.959）
D2-D3	0.018	0.130	0.891	（−0.244–0.280）
S + FNB Group				
D0-D1	1.213^a^	0.274	< 0.001	（0.660–1.765）
D0-D2	1.691^a^	0.141	< 0.001	（1.408–1.975）
D0-D3	1.702^a^	0.152	< 0.001	（1.397–2.008）
D1-D2	0.479^a^	0.158	< 0.05	（0.161–0.797）
D1-D3	0.489^a^	0.156	< 0.05	（0.175–0.804）
D2-D3	0.011	0.039	0.785	（−0.067–0.089）
S + LFCN Group				
D0-D1	1.075^a^	0.212	< 0.001	（0.648–1.503）
D0-D2	1.630^a^	0.200	< 0.001	（1.226–2.033）
D0-D3	1.653^a^	0.212	< 0.001	（1.226–2.080）
D1-D2	0.555^a^	0.159	< 0.05	（0.235–0.874）
D1-D3	0.578^a^	0.150	< 0.001	（0.275–0.880）
D2-D3	0.023	0.163	0.889	（−0.305–0.350）
S + FICB Group				
D0-D1	2.157^a^	0.183	< 0.001	（1.789–2.526）
D0-D2	2.350^a^	0.165	< 0.001	（2.019–2.682）
D0-D3	2.474^a^	0.171	< 0.001	（2.130–2.818）
D1-D2	0.193^a^	0.095	< 0.05	（0.003–0.384）
D1-D3	0.317^a^	0.082	< 0.001	（0.153–0.481）
D2-D3	0.124	0.090	0.175	（−0.057–0.305）

S Group, bupivacaine-fentanyl isobaric spinal anesthesia; S + FNB Group, spinal anesthesia + femoral nerve block; S + LFCN Group, spinal anesthesia + lateral femoral cutaneous nerve block; S + FICB Group, spinal anesthesia + fascia iliaca compartment block. D0, the day before surgery; D1, postoperative day 1; D2, postoperative day 2; D3, postoperative day 3.

a the significance level of the mean difference is 0.05.

**Table 4:** Simple effects analysis between groups in four groups of VAS scores.

Time	Mean difference	Standard error	*P*	95% confidence interval
D0				
S-S + FNB	−0.147	0.253	0.563	（−0.645–0.352）
S-S + LFCN	−0.218	0.251	0.387	（−0.714–0.278）
S-S + FICB	−0.239	0.251	0.342	（−0.735–0.257）
S + FNB-S + LFCN	−0.072	0.253	0.777	（−0.570–0.406）
S + FNB-S + FICB	−0.093	0.253	0.714	（−0.591–1.959）
S + LFCN-S + FICB	−0.021	0.251	0.933	（−0.517–0.475）
D1				
S-S + FNB	1.167^a^	0.153	<0.001	（0.866–1.468）
S-S + LFCN	0.957^a^	0.152	<0.001	（0.658–1.257）
S-S + FICB	2.018^a^	0.152	<0.001	（1.719–2.318）
S + FNB-S + LFCN	−0.209	0.153	0.172	（−0.510–0.092）
S + FNB-S + FICB	0.851^a^	0.153	<0.001	（0.550–1.152）
S + LFCN-S + FICB	1.061^a^	0.152	<0.001	（0.761–1.360）
D2				
S-S + FNB	0.007	0.138	0.958	（−0.264–0.279）
S-S + LFCN	−0.126	0.137	0.358	（−0.396–0.144）
S-S + FICB	0.573^a^	0.137	<0.001	（0.303–0.843）
S + FNB-S + LFCN	−0.133	0.138	0.334	（−0.405–0.138）
S + FNB-S + FICB	0.566^a^	0.138	<0.001	（0.295–0.837）
S + LFCN-S + FICB	0.699^a^	0.137	<0.001	（0.429–0.969）
D3				
S-S + FNB	−1.330E−5	0.121	1.000	（−0.239–0.239）
S-S + LFCN	−0.121	0.120	0.316	（−0.359–0.116）
S-S + FICB	0.679^a^	0.120	<0.001	（0.441–0.916）
S + FNB-S + LFCN	−0.121	0.121	0.319	（−0.360–0.118）
S + FNB-S + FICB	0.679^a^	0.121	<0.001	（0.440–0.918）
S + LFCN-S + FICB	0.800^a^	0.120	<0.001	（0.563–1.037）

S Group, bupivacaine-fentanyl isobaric spinal anesthesia; S + FNB Group, spinal anesthesia + femoral nerve block; S + LFCN Group, spinal anesthesia + lateral femoral cutaneous nerve block; S + FICB Group, spinal anesthesia + fascia iliaca compartment block. D0, the day before surgery; D1, postoperative day 1; D2, postoperative day 2; D3, postoperative day 3.

a The significance level of the mean difference is 0.05.

### 3.3 Secondary outcomes

#### 3.3.1 Comparison of postoperative complication rates

No statistically significant differences were observed among the four groups in the incidence of postoperative urinary retention, agitation, shivering, or dyspnea (*P* > 0.05). However, significant differences were identified in the incidence of postoperative nausea and vomiting and pruritus among the four groups (*P* < 0.05). Specifically, the incidence of postoperative nausea and vomiting and pruritus was significantly lower in group S + FICB than in group S (*P* < 0.05), whereas no significant differences were observed between the remaining groups (Table 5).

**Table 5:** Comparison of postoperative complications rates among the four groups.

Variable	S Group (*N* = 48)	S + FNB Group (*N* = 47)	S + LFCN Group (*N* = 48)	S + FICB Group (*N* = 48)	*χ^2^*	*P*
Nausea and vomiting	18（37.5）	15（31.9）	12（25.0）	6 (12.5)^a^	8.530	0.036
Pruritus	14（29.2）	12（25.5）	9（18.8）	3 (6.3)^a^	9.173	0.027
Urinary retention	4（8.3）	3（6.4）	3（6.3）	1 (2.1)	1.836	0.607
Agitation	7（14.6）	5（10.6）	4（8.3）	2 (4.2)	3.199	0.362
Shivering	2（4.2）	2（4.3）	1（2.1）	0 (0.0)	2.290	0.514
Dyspnea	1（20.0）	1（20.0）	1（20.0）	0 (0.0)	1.023	0.796

Data are presented as *N* (%), and compared using Chi-square test. S Group, bupivacaine-fentanyl isobaric spinal anesthesia; S + FNB Group, spinal anesthesia + femoral nerve block; S + LFCN Group, spinal anesthesia + lateral femoral cutaneous nerve block; S + FICB Group, spinal anesthesia + fascia iliaca compartment block. A *P* < 0.05 was considered statistically significant. Compared with Group S.

a *P* < 0.05.

#### 3.3.2 Comparison of postoperative pain management indicators

Statistically significant differences were observed among the four groups in the dose of sufentanil required for postoperative rescue analgesia (*P* < 0.05). The sufentanil dose required for rescue analgesia was significantly lower in group S + LFCN and group S + FICB than in group S (*P* < 0.05), and was lowest in group S + FICB, which required a significantly lower dose than groups S + FNB and S + LFCN (*P* < 0.05). Significant differences were also found in the number of patients requiring postoperative rescue analgesia among the four groups (*P* < 0.05). The number of patients requiring rescue analgesia was significantly lower in group S + FICB than in groups S + FNB, S + LFCN, and S (*P* < 0.05). In addition, postoperative analgesia satisfaction differed significantly among the four groups (*P* < 0.05). Postoperative satisfaction was significantly higher in group S + FICB than in group S (*P* < 0.05), whereas differences among the other groups were not statistically significant (Table 6).

**Table 6:** Comparison of postoperative pain management indicators among the four groups.

Variable	S Group (*N* = 48)	S + FNB Group (*N* = 47)	S + LFCN Group (*N* = 48)	S + FICB Group (*N* = 48)	*F/χ^2^*	*P*
The dose of sufentanil required for postoperative rescue analgesiaug(ug)	2.68 ± 0.16	2.58 ± 0.17	2.51 ± 0.18^c^	1.7 ± 0.35^a^^,^^b^^,^^c^	153.590	<0.001
The number of requiring rescue analgesia	19（39.6）	12（25.5）	11（22.9）	6（12.5)^a^^,^^b^^,^^c^	39.939	<0.001
Postoperative analgesia satisfaction					13.138	0.041
Satisfied	26（54.2）	33（70.2）	35（72.9）	41（85.4)^c^		
Acceptable	17（35.4）	10（21.3）	9（18.8）	7（14.6）		
Dissatisfied	5（10.4）	4（8.5）	4（8.3）	0（0）		

Data are presented as mean ± SD, and compared using One-way ANOVA; Data are presented as *N* (%), and compared using Chi-square test. S Group, bupivacaine-fentanyl isobaric spinal anesthesia; S + FNB Group, spinal anesthesia + femoral nerve block; S + LFCN Group, spinal anesthesia + lateral femoral cutaneous nerve block; S + FICB Group, spinal anesthesia + fascia iliaca compartment block. A *P* < 0.05 was considered statistically significant. Compared with Group S + FNB.

a *P* < 0.05; Compared with Group S + LFCN.

b *P* *<* 0.05; Compared with Group S.

c *P* *<* 0.05.

## 4 Discussion

This retrospective study demonstrated that isobaric spinal anesthesia alone or in combination with different nerve block techniques significantly improved perioperative pain control in elderly patients undergoing PFNA fixation. Among the anesthetic strategies evaluated, spinal anesthesia combined with fascia iliaca compartment block showed clear advantages over spinal anesthesia combined with femoral nerve block or lateral femoral cutaneous nerve block. Specifically, this combination provided superior analgesia on postoperative days 1, 2, and 3, reduced the dose of sufentanil required for postoperative rescue analgesia, decreased the number of patients requiring rescue analgesia, and significantly lowered the incidence of postoperative nausea, vomiting, and pruritus compared with isobaric spinal anesthesia alone.

Hip fractures and related surgical procedures often cause severe pain. Acute pain may lead to psychological excitation, anxiety, agitation, or even restlessness, and is considered an important contributing factor to the development of postoperative delirium in elderly patients with hip fractures (14). Previous studies have emphasized the importance of timely and effective pain control, as inadequately managed acute pain may gradually progress to long-term chronic pain, exerting substantial negative effects on patients (15). Therefore, reducing perioperative pain and postoperative complication rates in elderly patients with hip fractures is essential for improving postoperative recovery and overall quality of life. General anesthesia provides sufficient analgesia and muscle relaxation and facilitates anesthetic management; however, it is often associated with greater intraoperative hemodynamic fluctuations and an increased risk of postoperative respiratory complications (16). Spinal anesthesia, by contrast, has been widely used in femoral fracture surgery because of its lower incidence of systemic complications and minimal impact on postoperative cognitive function (17). Nevertheless, hyperbaric spinal anesthesia has a limited duration of blockade, which may be insufficient for major procedures such as hip surgery, and may induce demyelination-like lesions of the spinal cord. In clinical practice, direct subarachnoid puncture using a 25-G fine spinal needle has been shown to reduce the incidence of post–dural puncture headache without interfering with postoperative anticoagulation therapy, thereby decreasing the risk of lower-extremity venous thrombosis after orthopedic surgery. In addition, intrathecal administration of isobaric bupivacaine combined with fentanyl prolongs the duration of spinal blockade while exerting minimal effects on circulatory function in elderly patients (18, 19). However, patient positioning prior to isobaric spinal anesthesia, particularly the knee-flexed lateral decubitus position, may cause severe pain and reduce anesthetic comfort. Traditionally, intravenous opioids have been administered before anesthesia to alleviate pain during positioning, but this approach may result in opioid overdose and subsequent cardiovascular and respiratory depression (20). Peripheral nerve block techniques effectively interrupt nociceptive nerve transmission and, as localized anesthetic approaches, provide unilateral analgesia while minimizing adverse effects on respiratory and circulatory function. Preemptive nerve block analgesia can reduce severe pain induced by position changes during neuraxial anesthesia and facilitate postoperative recovery (21). Previous studies have reported that neuraxial anesthesia combined with lateral femoral cutaneous nerve block can reduce complications and pain compared with neuraxial anesthesia alone (22). Chan et al. demonstrated that neuraxial anesthesia combined with femoral nerve block provided effective analgesia, stable hemodynamics, and a low incidence of adverse events in patients undergoing knee arthroplasty (23). Wan et al. further suggested that neuraxial anesthesia combined with fascia iliaca compartment block offers effective analgesia and good safety profiles in patients undergoing total hip arthroplasty (24).

In the present study, VAS scores in group S were lower on postoperative days 2 and 3 than at 1 day preoperatively, whereas groups S + FNB, S + LFCN, and S + FICB exhibited significantly lower VAS scores on postoperative days 1, 2, and 3 compared with preoperative values. These findings indicate that isobaric spinal anesthesia alone and in combination with different nerve block techniques effectively reduce perioperative pain in elderly patients undergoing PFNA. On postoperative day 1, VAS scores in groups S + FNB, S + LFCN, and S + FICB were lower than those in group S, and postoperative satisfaction was higher in group S + FICB than in group S. These results suggest that combining spinal anesthesia with nerve block techniques provides superior early postoperative analgesia compared with spinal anesthesia alone. In this study, patients received intravenous sufentanil as rescue analgesia when postoperative pain control was inadequate. The dose of sufentanil required for rescue analgesia was significantly lower in groups S + LFCN and S + FICB than in group S, indicating that spinal anesthesia combined with nerve block techniques more effectively reduced perioperative pain and improved patient comfort. Notably, the fascia iliaca compartment block involves injection of local anesthetic into the potential space between the iliacus muscle and iliac fascia, allowing the anesthetic to spread within the compartment and block multiple nerves, including the femoral nerve, obturator nerve, and lateral femoral cutaneous nerve (25). A recent study showed that the FICB procedure did not block the obturator nerve (26), which may explain why FICB did not completely relieve the pain of hip fracture in patients. In the present study, VAS scores on postoperative days 1, 2, and 3 were significantly lower in group S + FICB than in groups S + FNB and S + LFCN, and both the dose of sufentanil required and the number of patients requiring rescue analgesia were significantly lower in group S + FICB. These findings further confirm that fascia iliaca compartment block provides superior analgesia compared with single-site femoral nerve block or lateral femoral cutaneous nerve block (24). Opioid-related adverse effects following intravenous analgesia mainly include nausea, vomiting, and respiratory depression (27). Previous studies have reported that fascia iliaca compartment block exerts a pronounced perioperative analgesic effect by blocking nociceptive transmission, maintaining perioperative hemodynamic stability, and reducing anesthesia-related complications (28). In the present study, the incidence of postoperative nausea, vomiting, and pruritus was significantly lower in group S + FICB than in group S, indicating that spinal anesthesia combined with fascia iliaca compartment block reduces postoperative opioid requirements and associated adverse effects in elderly patients undergoing PFNA. These findings are consistent with previous reports (29, 30).

Several limitations of this study should be acknowledged. First, residual confounding factors could not be completely eliminated. Published evidence confirms intrathecal fentanyl increases the risk of opioid-related pruritus and postoperative nausea and vomiting by stimulating spinal opioid receptors (31). In this study, 50 μg fixed-dose intrathecal fentanyl was a key confounding factor that interfered with the interpretation of postoperative pruritus and postoperative nausea and vomiting outcomes; In addition, since the anesthesia regimen was selected at the discretion of the attending anesthesiologist according to the patient's clinical condition, confounding by indication may exist: patients with higher preoperative pain risk or poorer physical status might be more likely to receive enhanced nerve-block strategies, which could potentially bias the comparison of postoperative pain outcomes. Second, this retrospective observational study enrolled consecutive eligible patients during the study period. Therefore, no prospective a *priori* sample size calculation was performed. *Post-hoc* statistical power analysis was conducted for the primary outcome of postoperative VAS scores using G*Power 3.1, with a significance level of *α* = 0.05, a target statistical power of 0.80, and a medium effect size of f = 0.25 for a four-group repeated-measures design, the theoretically required sample size was 187 participants. The final sample of 191 patients in the present study exceeded this theoretical requirement, yielding an actual statistical power greater than 0.80 and providing sufficient power to detect group and temporal differences in postoperative VAS scores. Nevertheless, owing to the retrospective nature and *post-hoc* design, a potential risk of type II error cannot be completely excluded. Third, this study was unable to collect operating diagrams and ultrasound images during the nerve block in each group. Fourth, no severe adverse events were recorded in this study, which may be attributable to recall bias during postoperative follow-up, as many patients did not remember mild or moderate adverse events. Fifth, absence of long-term follow-up (chronic pain transition), and single-center design limiting generalizability. Therefore, large-scale, multicenter prospective studies with more detailed clinical data are warranted to identify the optimal analgesic strategy for elderly patients undergoing PFNA.

In conclusion, in elderly patients undergoing proximal femoral nail antirotation, preoperative isobaric spinal anesthesia combined with fascia iliaca compartment block was associated with improved postoperative pain, reduced the incidence of adverse reactions, improved patient comfort, and was more effective than other anesthesia methods.

## Data Availability

The original contributions presented in the study are included in the article/Supplementary Material, further inquiries can be directed to the corresponding author.
